# Upper Bounds for the Capacity for Severely Fading MIMO Channels under a Scale Mixture Assumption

**DOI:** 10.3390/e23070845

**Published:** 2021-06-30

**Authors:** Johannes T. Ferreira

**Affiliations:** 1 Department of Statistics, University of Pretoria, Pretoria 0002, South Africa; johan.ferreira@up.ac.za; 2 Centre of Excellence in Mathematical and Statistical Science, University of the Witwatersrand, Johannesburg 2000, South Africa

**Keywords:** ergodic capacity, eigenvalues, noncentrality

## Abstract

A cornerstone in the modeling of wireless communication is MIMO systems, where a complex matrix variate normal assumption is often made for the underlying distribution of the propagation matrix. A popular measure of information, namely capacity, is often investigated for the performance of MIMO designs. This paper derives upper bounds for this measure of information for the case of two transmitting antennae and an arbitrary number of receiving antennae when the propagation matrix is assumed to follow a scale mixture of complex matrix variate normal distribution. Furthermore, noncentrality is assumed to account for LOS scenarios within the MIMO environment. The insight of this paper illustrates the theoretical form of capacity under these key assumptions and paves the way for considerations of alternative distributional choices for the channel propagation matrix in potential cases of severe fading, when the assumption of normality may not be realistic.

## 1. Introduction

### 1.1. MIMO Channels and Capacity

Research in random matrix theory has seen unprecedented growth in not only theoretical advances over the last two decades, but also coupled with a variety of developments in fields of application including wireless communication. In fact, the development in this current era of growing need for continuous and more reliable communication motivates a sustained research interest in mathematical, and specifically statistical representation and theoretical frameworks to understand and extend global communication needs. A major consideration within wireless communication is the multiple-input-multiple-output (MIMO) design. MIMO designs are widely studied and employed in practice as their multiple-antenna design exhibits high spectral efficiency and increased capacity for the transfer of information via wireless signals passing through these systems. MIMO has a fundamental probabilistic foundation, and is intertwined with statistical distribution theory. Therefore, statistical distribution insight is essential for further development, refinement, and investigation of MIMO systems and the interconnected world we live and operate in.

In wireless communication, this design is characterized as y=Hx+e where $y,e\in C^{n\times 1}$, $x\in C^{p\times 1}$, and $H\in C^{n\times p}$. In MIMO terms, x and y denotes the transmit- and receiver signal vectors respectively, e denotes a noise vector, and *n* denotes the number of transmitter antennae and *p* denotes the number of receiver antennae (often denoted by $n_{t}$ and $n_{r}$ respectively—however, "usual" statistical notation of *n* and *p* will be retained). Here, $C^{n\times p}$ denotes the space of all complex matrices of dimension n×p and $C_{*}^{p\times p}$ denotes the space of all Hermitian positive definite complex matrices of dimension p×p. H is called the propagation matrix, and is usually considered to be complex matrix variate normally distributed with probability density function (pdf)

(1) $$f\left(H\right)=\frac{1}{\pi^{np}\det{\left(\Phi \right)}^{p}\det{\left(\Sigma \right)}^{n}}etr\left(-\Phi^{-1}\left(H-M\right)\Sigma^{-1}{\left(H-M\right)}^{H}\right)$$

denoted $H∼CN_{n\times p}\left(M,\Phi ⊗\Sigma \right)$, where $M\in C^{n\times p}$, $\Phi \in C_{*}^{n\times n}$, $\Sigma \in C_{*}^{p\times p}$ with mean $E\left(H\right)=M$ and $cov\left(H\right)=\Phi ⊗\Sigma$. $X^{H}$, $X^{-1}$, and det(X) denotes the Hermitian transpose, the inverse, and the determinant of the complex matrix X respectively, ⊗ denotes the Kronecker product, $etr\left(\cdot \right)$ denotes the exponential trace $\exp\left(tr\cdot \right)$, and $E\left(\cdot \right)$ and $cov\left(\cdot \right)$ denotes the expectation and covariance operator.

MIMO systems have different measures of information that describes the efficacy and quality of transmitted information in the design: mutual information being an important characteristic. In particular, the ergodic mutual information of the MIMO channel is evaluated as (see [1])

(2) $$\begin{array}{ccc} C & = & E_{H}\left(\log\det\left(I_{p}+\frac{\rho }{p}H^{H}H\right)\right)\\ & = & pE_{H}\left(\log\left(1+\frac{\rho }{p}\lambda_{1}\right)\right) \end{array}$$

where ρ denotes the signal-to-noise ratio, and $I_{p}$ denotes the identity matrix of dimension *p*. The measure of information (2) is often described as the capacity of the MIMO system (also called Shannon capacity, see [2]), and relies on the distribution of $H^{H}H$ - or what is known as the complex (noncentral) Wishart matrix when H is considered CN distributed. Here, $\lambda_{1}$ denotes the largest eigenvalue of $H^{H}H$, and its distribution is essential in the evaluation of (2). This assumption for H is restrictive in the practical case when severe fading is observed, and it is the main focus of this paper to alleviate this restriction via the platform of scale mixture of complex matrix variate normals for H.

### 1.2. Scale Mixture of (Complex) Matrix Variate Normals and the Resulting Wishart

Normality remains a common and useful assumption within statistical theory- and application. However, data often display characteristics that do not align with the implied requirements of normality: characteristics such as heavier tails, even if the data still respects characteristics such as symmetry. The alleviation of this normal assumption has been the focus of much research. Two important research foci in this regard are Gaussian mixtures (see [3,4] for examples in the MIMO context) which can account for multimodality and skewness, and scale mixtures which can account for heavier-than-normal tails in the data. Scale mixtures are often considered for relaxing the normal assumption, and is a particular focus area of this paper. Pioneering work has been undertaken by [5,6,7] and has brought forth an important wave of research since due to the elegance and practicality of this broader elliptical class of models. In the complex variate realm, [8] capitalized on this mixture representation of complex normal variates. However, limited work has been done to introduce the platform of scale mixture of normal distributions within a MIMO context. Within MIMO, a normal assumption is made, but evidence exists that there are practical considerations from fieldwork which supports the argument that a departure from normality does not seem far-fetched [9,10,11,12]. In this light, the consideration of a scale mixture of (complex) matrix variate normals (SMCN) for the candidacy of H makes a meaningful contribution, as the scale mixture class has different distributional members which may very well suitably adapt to the practitioners need [9,13]. These members include the usual normal-, t-, contaminated normal-, and slash distribution among others—all of which provide a heavier-than-normal tailed alternative for potential practical considerations of H. In fact, [11] illustrates an SMCN assumption for a zero mean H in the MIMO context specifically where superior capacity performance is observed for an underlying complex matrix variate t model for H, and [10,12] demonstrates the added value of the scale mixture approach when H exhibits a nonzero mean; specifically focusing on rank-1 noncentrality and the condition number of the quadratic form, respectively. The data-driven consideration of the scale mixture approach in the MIMO environment is therefore well-motivated, and a valuable theoretical consideration.

The variable H has an SMCN distribution if it has pdf

(3) $$\begin{array}{ccc} f(H) & = & \frac{1}{\det{\left(\Phi \right)}^{p}\det{\left(\Sigma \right)}^{n}}h\left(-tr\Phi^{-1}\left(H-M\right)\Sigma^{-1}{\left(H-M\right)}^{H}\right)\\ & = & \int_{R^{+}}f_{CN_{n\times p}\left(M,\Phi ⊗t^{-1}\Sigma \right)}\left(H|t\right)k\left(t\right)dt \end{array}$$

denoted by $H∼SMCN_{n\times p}\left(M,\Phi ⊗\Sigma ,h\right)$ where $f_{CN_{n\times p}\left(M,\Phi ⊗t^{-1}\Sigma \right)}$ denotes the CN pdf as in (1), $h\left(\cdot \right)$ denotes the generator function, and $k\left(t\right)$ denotes the weight function (inherently dependent on $h\left(\cdot \right)$). The advantage of this mixture representation is that the variable $H∼SMCN_{n\times p}\left(M,\Phi ⊗\Sigma ,h\right)$ retains key statistical characteristics $E\left(H\right)=M$ and $cov\left(H\right)=\Phi ⊗\Sigma$. There are two aspects of (3) that warrants some comments for this paper: first, the assumption of a nonzero mean (in this case M) which often causes a challenging theoretical environment in which to investigate properties of the model. Secondly, the large covariance structure which causes further theoretical challenges, where the trace operator in the argument of $h\left(\cdot \right)$ oftentimes restrict computable forms. Key characteristics under these challenges (such as pdfs of the joint eigenvalues, and thus capacity) are often derived in mathematically elegant yet computationally challenging forms of zonal- and Hayakawa polynomials (see [2], for example). This limits the practical consideration, and this paper will make additional assumptions (similar to [10,14,15]), such as $\Phi =I_{n}$ to circumvent the cumbersome potential computational implementation of the results.

### 1.3. Main Contribution of this Paper

The main contribution in this study is assuming this SMCN distributional form (3) for H, compared to the usual complex normal assumption (1). This results in the distribution of $S=H^{H}H\in C_{*}^{p\times p}$ (see [10]), necessary for deriving (2), with pdf

(4) $$f\left(S\right)=\frac{{\left(\det S\right)}^{n-p}}{{\tilde{\Gamma }}_{p}\left(n\right)\det{\left(\Sigma \right)}^{n}}\int_{R^{+}}t^{np}etr\left(-t\left(\Sigma^{-1}S+\Delta \right)\right){\;}_{0}{\tilde{F}}_{1}\left(n;t^{2}\Delta \Sigma^{-1}S\right)k\left(t\right)dt$$

denoted by $S∼SMCW_{p}\left(n,M,I_{n}⊗\Sigma \right)$ where $S\in C_{*}^{p\times p}$, $\Delta =\Sigma^{-1}M^{H}M$ indicates the noncentral matrix parameter, and ${\tilde{\Gamma }}_{p}\left(\cdot \right)$ denotes the p−dimensional complex multivariate gamma function. The function ${}_{r}{\tilde{F}}_{q}\left(a_{1},...,a_{r};b_{1},...,b_{q},X\right)$ denotes the hypergeometric function of Hermitian matrix argument with *r* upper and *q* lower parameters (see [2,16] for further details) where r=0 and q=1 in (4) specifically. The distribution in (4) is called a scale mixture of complex Wishart (SMCW) and will be referred to as Case 1, where the consideration of the parameter Δ implies noncentrality. [17] (and subsequently [12]) also considered an uncorrelated SMCW model within the MIMO environment. This consideration simplifies the theoretical framework, but is practically motivated for spatial (in)dependence of transmitters for when antennae are spaced far enough apart geographically so that the assumption of independence is not unreasonable. Consider thus the transformation $W=\Sigma^{-1}S\in C_{*}^{p\times p}$ with Jacobian $J\left(S\to W\right)=\det{\left(\Sigma \right)}^{p}$, then (see [12])

(5) $$f_{unc}\left(W\right)=\frac{{\left(\det W\right)}^{n-p}}{{\tilde{\Gamma }}_{p}\left(n\right)}\int_{R^{+}}t^{np}etr\left(-t\left(W+\Delta \right)\right){\;}_{0}{\tilde{F}}_{1}\left(n;t^{2}\Delta W\right)k\left(t\right)dt$$

and will be referred to as Case 2.

The second main consideration of this paper is assuming a nonzero mean matrix for H. This is a valid and oftentimes essential assumption for a MIMO system when there is a direct line-of-sight (LOS) component between transmitters and receivers (see [10]). This noncentrality is also a fundamental component in expressions left in zonal- and Hayakawa polynomial form. To this effect, expressions which are effectively an upper bound for the capacity are derived under the SMCN assumption, assuming noncentrality for H. Together, these two considerations will give insight into the (comparatively) little researched phenomena into severely fading channels and accounting for LOS between transmitters and receivers (see [1] as well as the Appendix A).

For observing the capacity, the joint pdf of the eigenvalues of (4) and (5) are derived; however, the form of these pdfs are challenging to work with for arbitrary *n* and *p*. The assumptions of this paper as well as the derivation of an upper bound for Case 1 and 2 will ease this investigation to allow clearer insight into the conceptual behavior in the case of a SMCN assumption H. The case when p=2 will be of particular illustrative interest, for both Cases 1 and 2. This way, a contribution is also made to allow for arbitrary *n*: arbitrary degrees of freedom for the SMCW distributions under consideration in this paper, and arbitrary number of transmitter antennae in the MIMO environment.

The outline of this paper is as follows: in Section 2, the joint pdfs of the eigenvalues for both Case 1 and Case 2 are derived, and a specific upper bound for these densities are derived. These expressions are used in Section 3 to derive an upper bound for the capacity for Case 1 and Case 2. A particular exact expression for Case 2 is also given. Section 4 contains a discussion of obtained results and some potential areas for further work.

## 2. Eigenvalue Pdfs and an Upper Bound

As the eigenvalues of S (and W) respectively play an essential role in understanding the capacity of a MIMO system subjected to this alternate distributional consideration for H, determining the joint pdf of the eigenvalues of S (and W) are of interest. Suppose that $\lambda_{1}>\lambda_{2}>...>\lambda_{p}>0$ denote the real ordered eigenvalues of S, and let $\Lambda =diag\left(\lambda_{1},\lambda_{2},...,\lambda_{p}\right)$. Additionally, let $\mu_{1}>\mu_{2}>...>\mu_{p}>0$ denote the real ordered eigenvalues of Δ, and let $\Omega =diag\left(\mu_{1},\mu_{2},...,\mu_{p}\right)$. The joint pdf of Λ is then given by [18])

(6) $$f\left(\Lambda \right)=\frac{\pi^{p\left(p-1\right)}}{{\tilde{\Gamma }}_{p}\left(p\right)}\prod_{k<l}^{p}{\left(\lambda_{k}-\lambda_{l}\right)}^{2}\int_{E\in U\left(p\right)}f\left(E\Lambda E^{H}\right)dE$$

where the pdf $f\left(\cdot \right)$ in the integral in (6) denotes the pdf of S, and $U\left(p\right)$ denotes the unitary space of order *p* (see [18]). For Case 1 (see (4)) the joint pdf of Λ is then given by [10] (Note that $\prod_{k<l}^{p}\left(\lambda_{k}-\lambda_{l}\right)$ is often referred to as the Vandermonde determinant.)

(7) $$\begin{array}{ccc} f\left(\Lambda \right) & = & \frac{\pi^{p\left(p-1\right)}\det{\left(\Lambda \right)}^{n-p}}{{\tilde{\Gamma }}_{p}\left(p\right){\tilde{\Gamma }}_{p}\left(n\right)\det{\left(\Sigma \right)}^{n}}\prod_{k<l}^{p}{\left(\lambda_{k}-\lambda_{l}\right)}^{2}\int_{R^{+}}t^{np}etr\left(-t\Delta \right)\\ & & \times \int_{E\in U\left(p\right)}etr\left(-t\Sigma^{-1}E\Lambda E^{H}\right){\;}_{0}{\tilde{F}}_{1}\left(n;t^{2}\Delta \Sigma^{-1}E\Lambda E^{H}\right)dEk\left(t\right)dt. \end{array}$$

This equation, in terms of an integral over the unitary space, leads to a nonpractical representation of the joint pdf of the eigenvalues. Ref. [2] proved the following upper bound for $X\in C^{n\times p}$:

(8) $${}_{0}{\tilde{F}}_{1}\left(n;X^{H}X\right)\leq_{\;0}{\tilde{F}}_{0}\left(\frac{X^{H}X}{n}\right)=etr\left(\frac{X^{H}X}{n}\right)$$

and illustrated the tightness of this upper bound. Thus, to circumvent the theoretical challenge in (8) we apply (8) to obtain an upper bound for this joint pdf, and use the splitting formula of [18]:

(9) $$\begin{array}{ccc} & & f\left(\Lambda \right)\\ & \leq & \frac{\pi^{p\left(p-1\right)}\det{\left(\Lambda \right)}^{n-p}}{{\tilde{\Gamma }}_{p}\left(p\right){\tilde{\Gamma }}_{p}\left(n\right)\det{\left(\Sigma \right)}^{n}}\prod_{k<l}^{p}{\left(\lambda_{k}-\lambda_{l}\right)}^{2}\int_{R^{+}}t^{np}etr\left(-t\Delta \right)\\ & & \times \int_{E\in U\left(p\right)}etr\left(-t\Sigma^{-1}E\Lambda E^{H}\right){\;}_{0}{\tilde{F}}_{0}\left(\frac{t^{2}\Delta \Sigma^{-1}E\Lambda E^{H}}{n}\right)dEk\left(t\right)dt\\ & = & \frac{\pi^{p\left(p-1\right)}\det{\left(\Lambda \right)}^{n-p}}{{\tilde{\Gamma }}_{p}\left(p\right){\tilde{\Gamma }}_{p}\left(n\right)\det{\left(\Sigma \right)}^{n}}\prod_{k<l}^{p}{\left(\lambda_{k}-\lambda_{l}\right)}^{2}\int_{R^{+}}t^{np}etr\left(-t\Delta \right)\\ & & \times \int_{E\in U\left(p\right)}etr\left(-\left(t\Sigma^{-1}-\frac{t^{2}\Delta \Sigma^{-1}}{n}\right)E\Lambda E^{H}\right)dEk\left(t\right)dt\\ & = & \frac{\pi^{p\left(p-1\right)}\det{\left(\Lambda \right)}^{n-p}}{{\tilde{\Gamma }}_{p}\left(p\right){\tilde{\Gamma }}_{p}\left(n\right)\det{\left(\Sigma \right)}^{n}}\prod_{k<l}^{p}{\left(\lambda_{k}-\lambda_{l}\right)}^{2}\int_{R^{+}}t^{np}etr\left(-t\Delta \right){\;}_{0}{\tilde{F}}_{0}^{\left(p\right)}\left(-\Psi ,\Lambda \right)k\left(t\right)dt \end{array}$$

where $\Psi =t\Sigma^{-1}-\frac{t^{2}\Delta \Sigma^{-1}}{n}$ and ${}_{0}{\tilde{F}}_{0}^{\left(p\right)}\left(-\Psi ,\Lambda \right)$ denotes the hypergeometric of double complex matrix argument [18]. For Case 2 (see (5)), in a similar way as above, this pdf (denoted by $f_{unc}\left(\cdot \right)$ for Case 2) is given by

(10) $$\begin{array}{ccc} & & f_{unc}\left(\Lambda \right)\\ & = & \frac{\pi^{p\left(p-1\right)}\det{\left(\Lambda \right)}^{n-p}}{{\tilde{\Gamma }}_{p}\left(p\right){\tilde{\Gamma }}_{p}\left(n\right)}\prod_{k<l}^{p}{\left(\lambda_{k}-\lambda_{l}\right)}^{2}\int_{R^{+}}t^{np}etr\left(-t\Delta \right)\\ & & \times \int_{E\in U\left(p\right)}etr\left(-tE\Lambda E^{H}\right){\;}_{0}{\tilde{F}}_{1}\left(n;t^{2}\Delta E\Lambda E^{H}\right)dEk\left(t\right)dt\\ & \leq & \frac{\pi^{p\left(p-1\right)}\det{\left(\Lambda \right)}^{n-p}}{{\tilde{\Gamma }}_{p}\left(p\right){\tilde{\Gamma }}_{p}\left(n\right)}\prod_{k<l}^{p}{\left(\lambda_{k}-\lambda_{l}\right)}^{2}\int_{R^{+}}t^{np}etr\left(-t\Delta \right)\\ & & \times \int_{E\in U\left(p\right)}etr\left(-tE\Lambda E^{H}\right){\;}_{0}{\tilde{F}}_{0}\left(\frac{t^{2}\Delta E\Lambda E^{H}}{n}\right)dEk\left(t\right)dt\\ & = & \frac{\pi^{p\left(p-1\right)}\det{\left(\Lambda \right)}^{n-p}}{{\tilde{\Gamma }}_{p}\left(p\right){\tilde{\Gamma }}_{p}\left(n\right)}\prod_{k<l}^{p}{\left(\lambda_{k}-\lambda_{l}\right)}^{2}\int_{R^{+}}t^{np}etr\left(-t\Delta \right){\;}_{0}{\tilde{F}}_{0}^{\left(p\right)}\left(-\Psi_{*},\Lambda \right)k\left(t\right)dt \end{array}$$

where $\Psi_{*}=tI_{p}-\frac{t^{2}\Delta }{n}$. A useful representation of ${}_{0}{\tilde{F}}_{0}^{\left(p\right)}\left(-\Psi ,\Lambda \right)$ in (10) (and ${}_{0}{\tilde{F}}_{0}^{\left(p\right)}\left(-\Psi_{*},\Lambda \right)$ in (11)) is given by [16]:

(11) $${}_{0}{\tilde{F}}_{0}^{\left(p\right)}\left(-\Psi ,\Lambda \right)=\frac{{\tilde{\Gamma }}_{p}\left(p\right)\det\left(\exp\left(-\psi_{i}\lambda_{j}\right)\right)}{\pi^{\frac{p\left(p-1\right)}{2}}\prod_{k<l}^{p}\left(\lambda_{k}-\lambda_{l}\right)\prod_{k<l}^{p}\left(\psi_{k}-\psi_{l}\right)}$$

where $\psi_{1}>\psi_{2}>...>\psi_{p}>0$ denotes the real ordered eigenvalues of Ψ (and $\psi_{1,*}>\psi_{2,*}>...>\psi_{p,*}>0$ denotes the real ordered eigenvalues of $\Psi_{*}$). Using (11) and obtaining the unordered joint pdf of the eigenvalues (see [19]) leaves

(12) $$\begin{array}{ccc} & & f\left(\lambda_{1},\lambda_{2},...,\lambda_{p}\right)\\ & \leq & \frac{\pi^{p\left(p-1\right)}\det{\left(\Lambda \right)}^{n-p}}{p!{\tilde{\Gamma }}_{p}\left(p\right){\tilde{\Gamma }}_{p}\left(n\right)\det{\left(\Sigma \right)}^{n}}\prod_{k<l}^{p}{\left(\lambda_{k}-\lambda_{l}\right)}^{2}\int_{R^{+}}t^{np}etr\left(-t\Delta \right)\\ & & \times \;\frac{{\tilde{\Gamma }}_{p}\left(p\right)\det\left(\exp\left(-\psi_{i}\lambda_{j}\right)\right)}{\pi^{\frac{p\left(p-1\right)}{2}}\prod_{k<l}^{p}\left(\lambda_{k}-\lambda_{l}\right)\prod_{k<l}^{p}\left(\psi_{k}-\psi_{l}\right)}k\left(t\right)dt\\ & = & \frac{\pi^{\frac{p\left(p-1\right)}{2}}\det{\left(\Lambda \right)}^{n-p}}{p!{\tilde{\Gamma }}_{p}\left(n\right)\det{\left(\Sigma \right)}^{n}}\prod_{k<l}^{p}\left(\lambda_{k}-\lambda_{l}\right)\\ & & \times \int_{R^{+}}t^{np}\frac{etr\left(-t\Delta \right)}{\prod_{k<l}^{p}\left(\psi_{k}-\psi_{l}\right)}\det\left(\exp\left(-\psi_{i}\lambda_{j}\right)\right)k\left(t\right)dt \end{array}$$

and

(13) $$\begin{array}{ccc} & & f_{unc}\left(\lambda_{1},\lambda_{2},...,\lambda_{p}\right)\\ & \leq & \frac{\pi^{p\left(p-1\right)}\det{\left(\Lambda \right)}^{n-p}}{p!{\tilde{\Gamma }}_{p}\left(p\right){\tilde{\Gamma }}_{p}\left(n\right)}\prod_{k<l}^{p}{\left(\lambda_{k}-\lambda_{l}\right)}^{2}\int_{R^{+}}t^{np}etr\left(-t\Delta \right)\\ & & \times \;\frac{{\tilde{\Gamma }}_{p}\left(p\right)\det\left(\exp\left(-\psi_{i,*}\lambda_{j}\right)\right)}{\pi^{\frac{p\left(p-1\right)}{2}}\prod_{k<l}^{p}\left(\lambda_{k}-\lambda_{l}\right)\prod_{k<l}^{p}\left(\psi_{k,*}-\psi_{l,*}\right)}k\left(t\right)dt\\ & = & \frac{\pi^{\frac{p\left(p-1\right)}{2}}\det{\left(\Lambda \right)}^{n-p}}{p!{\tilde{\Gamma }}_{p}\left(n\right)}\prod_{k<l}^{p}\left(\lambda_{k}-\lambda_{l}\right)\\ & & \times \int_{R^{+}}t^{np}\frac{etr\left(-t\Delta \right)}{\prod_{k<l}^{p}\left(\psi_{k,*}-\psi_{l,*}\right)}\det\left(\exp\left(-\psi_{i,*}\lambda_{j}\right)\right)k\left(t\right)dt. \end{array}$$

There are clear theoretical challenges in using (8) or (10), or even (10) or (11) in the quest to determine explicit expressions for the capacity (see (2)). Using (13) and (14), an upper bound can now be obtained for the capacity using the marginal distribution for $\lambda_{1}$ in each respective case.

## 3. Capacity for the Case p=2

In this section, expressions for an upper bound for the capacity in (2) when assuming an SMCN for H is derived when p=2 for Case 1 and 2. An exact expression for *C* is also derived for Case 2.

### 3.1. Approximation for Case 1 and Case 2

Considering Case 1 (13) and setting p=2, see that

(14) $$\begin{array}{ccc} \det\left(\exp\left(-\psi_{i}\lambda_{j}\right)\right) & = & \left|\begin{array}{cc} \exp\left(-\psi_{1}\lambda_{1}\right) & \exp\left(-\psi_{1}\lambda_{2}\right)\\ \exp\left(-\psi_{2}\lambda_{1}\right) & \exp\left(-\psi_{2}\lambda_{2}\right) \end{array}\right|\\ & = & \exp\left(-\psi_{1}\lambda_{1}-\psi_{2}\lambda_{2}\right)-\exp\left(-\psi_{1}\lambda_{2}-\psi_{2}\lambda_{1}\right). \end{array}$$

Therefore, by substituting (15) into (13) and determining (2):

(15) $$\begin{array}{ccc} C & \leq & \int_{R^{+}}2\log\left[1+\frac{\rho }{2}\lambda_{1}\right]\int_{R^{+}}\frac{\pi {\left(\lambda_{1}\lambda_{2}\right)}^{n-2}\left(\lambda_{1}-\lambda_{2}\right)}{2!{\tilde{\Gamma }}_{2}\left(n\right)\det{\left(\Sigma \right)}^{n}}\\ & & \times \int_{R^{+}}t^{2n}\frac{etr\left(-t\Delta \right)}{\left(\psi_{1}-\psi_{2}\right)}\left(\exp\left(-\psi_{1}\lambda_{1}-\psi_{2}\lambda_{2}\right)-\exp\left(-\psi_{1}\lambda_{2}-\psi_{2}\lambda_{1}\right)\right)k\left(t\right)dtd\lambda_{2}d\lambda_{1}\\ & = & \frac{1}{\Gamma \left(n\right)\Gamma \left(n-1\right)\det{\left(\Sigma \right)}^{n}}\int_{R^{+}}\log\left[1+\frac{\rho }{2}\lambda_{1}\right]\int_{R^{+}}t^{2n}\frac{etr\left(-t\Delta \right)}{\left(\psi_{1}-\psi_{2}\right)}\\ & & \times \int_{R^{+}}\left(\lambda_{1}^{n-1}\lambda_{2}^{n-2}-\lambda_{1}^{n-2}\lambda_{2}^{n-1}\right)\\ & & \times \left(\exp\left(-\psi_{1}\lambda_{1}-\psi_{2}\lambda_{2}\right)-\exp\left(-\psi_{1}\lambda_{2}-\psi_{2}\lambda_{1}\right)\right)d\lambda_{2}k\left(t\right)dtd\lambda_{1}. \end{array}$$

Using the gamma integral relation (see [20], p. 337), see that

(16) $$\begin{array}{ccc} & & \int_{R^{+}}\left(\lambda_{1}^{n-1}\lambda_{2}^{n-2}-\lambda_{1}^{n-2}\lambda_{2}^{n-1}\right)\left(\exp\left(-\psi_{1}\lambda_{1}-\psi_{2}\lambda_{2}\right)-\exp\left(-\psi_{1}\lambda_{2}-\psi_{2}\lambda_{1}\right)\right)d\lambda_{2}\\ & = & \lambda_{1}^{n-1}\exp\left(-\psi_{1}\lambda_{1}\right)\psi_{2}^{-\left(n-1\right)}\Gamma \left(n-1\right)-\lambda_{1}^{n-1}\exp\left(-\psi_{2}\lambda_{1}\right)\psi_{1}^{-\left(n-1\right)}\Gamma \left(n-1\right)\\ & & -\lambda_{1}^{n-2}\exp\left(-\psi_{1}\lambda_{1}\right)\psi_{2}^{-n}\Gamma \left(n\right)+\lambda_{1}^{n-2}\exp\left(-\psi_{2}\lambda_{1}\right)\psi_{1}^{-n}\Gamma \left(n\right). \end{array}$$

Substituting (16) into (16) leaves

$$\begin{array}{ccc} C & \leq & \frac{1}{\Gamma \left(n\right)\Gamma \left(n-1\right)\det{\left(\Sigma \right)}^{n}}\int_{R^{+}}\int_{R^{+}}\log\left[1+\frac{\rho }{2}\lambda_{1}\right]t^{2n}\frac{etr\left(-t\Delta \right)}{\left(\psi_{1}-\psi_{2}\right)}\\ & & \times (\lambda_{1}^{n-1}\exp\left(-\psi_{1}\lambda_{1}\right)\psi_{2}^{-\left(n-1\right)}\Gamma \left(n-1\right)-\lambda_{1}^{n-1}\exp\left(-\psi_{2}\lambda_{1}\right)\psi_{1}^{-\left(n-1\right)}\Gamma \left(n-1\right)\\ & & -\lambda_{1}^{n-2}\exp\left(-\psi_{1}\lambda_{1}\right)\psi_{2}^{-n}\Gamma \left(n\right)+\lambda_{1}^{n-2}\exp\left(-\psi_{2}\lambda_{1}\right)\psi_{1}^{-n}\Gamma \left(n\right))k\left(t\right)dtd\lambda_{1}\\ & = & \frac{1}{\det{\left(\Sigma \right)}^{n}}[\int_{R^{+}}t^{2n}\frac{etr\left(-t\Delta \right)\psi_{2}^{-\left(n-1\right)}}{\Gamma \left(n\right)\left(\psi_{1}-\psi_{2}\right)}\int_{R^{+}}\log\left[1+\frac{\rho }{2}\lambda_{1}\right]\lambda_{1}^{n-1}\exp\left(-\psi_{1}\lambda_{1}\right)d\lambda_{1}k\left(t\right)dt\\ & & -\int_{R^{+}}t^{2n}\frac{etr\left(-t\Delta \right)\psi_{1}^{-\left(n-1\right)}}{\Gamma \left(n\right)\left(\psi_{1}-\psi_{2}\right)}\int_{R^{+}}\log\left[1+\frac{\rho }{2}\lambda_{1}\right]\lambda_{1}^{n-1}\exp\left(-\psi_{2}\lambda_{1}\right)d\lambda_{1}k\left(t\right)dt\\ & & -\int_{R^{+}}t^{2n}\frac{etr\left(-t\Delta \right)\psi_{2}^{-n}}{\Gamma \left(n-1\right)\left(\psi_{1}-\psi_{2}\right)}\int_{R^{+}}\log\left[1+\frac{\rho }{2}\lambda_{1}\right]\lambda_{1}^{n-2}\exp\left(-\psi_{1}\lambda_{1}\right)d\lambda_{1}k\left(t\right)dt\\ & & +\int_{R^{+}}t^{2n}\frac{etr\left(-t\Delta \right)\psi_{1}^{-n}}{\Gamma \left(n-1\right)\left(\psi_{1}-\psi_{2}\right)}\int_{R^{+}}\log\left[1+\frac{\rho }{2}\lambda_{1}\right]\lambda_{1}^{n-2}\exp\left(-\psi_{2}\lambda_{1}\right)d\lambda_{1}k\left(t\right)dt]. \end{array}$$

For Case 2 (14), the result would follow in a similar manner:

$$\begin{array}{ccc} C & \leq & \int_{R^{+}}t^{2n}\frac{etr\left(-t\Delta \right)\psi_{2,*}^{-\left(n-1\right)}}{\Gamma \left(n\right)\left(\psi_{1,*}-\psi_{2,*}\right)}\int_{R^{+}}\log\left[1+\frac{\rho }{2}\lambda_{1}\right]\lambda_{1}^{n-1}\exp\left(-\psi_{1,*}\lambda_{1}\right)d\lambda_{1}k\left(t\right)dt\\ & & -\int_{R^{+}}t^{2n}\frac{etr\left(-t\Delta \right)\psi_{1,*}^{-\left(n-1\right)}}{\Gamma \left(n\right)\left(\psi_{1,*}-\psi_{2,*}\right)}\int_{R^{+}}\log\left[1+\frac{\rho }{2}\lambda_{1}\right]\lambda_{1}^{n-1}\exp\left(-\psi_{2,*}\lambda_{1}\right)d\lambda_{1}k\left(t\right)dt\\ & & -\int_{R^{+}}t^{2n}\frac{etr\left(-t\Delta \right)\psi_{2,*}^{-n}}{\Gamma \left(n-1\right)\left(\psi_{1,*}-\psi_{2,*}\right)}\int_{R^{+}}\log\left[1+\frac{\rho }{2}\lambda_{1}\right]\lambda_{1}^{n-2}\exp\left(-\psi_{1,*}\lambda_{1}\right)d\lambda_{1}k\left(t\right)dt\\ & & +\int_{R^{+}}t^{2n}\frac{etr\left(-t\Delta \right)\psi_{1,*}^{-n}}{\Gamma \left(n-1\right)\left(\psi_{1,*}-\psi_{2,*}\right)}\int_{R^{+}}\log\left[1+\frac{\rho }{2}\lambda_{1}\right]\lambda_{1}^{n-2}\exp\left(-\psi_{2,*}\lambda_{1}\right)d\lambda_{1}k\left(t\right)dt. \end{array}$$

Both these approximations for Case 1 and 2 illustrate the elegance which the scale mixture platform provides in a theoretical sense, and under the usual CN assumption (with corresponding weight k(t)) the results reflect those obtained by [2]. In the zero mean case, i.e., when M=0 and thus Λ=0, the results of [11] are closely reflected. A computational challenge remains due to the dependence of these expressions on $\psi_{1},\psi_{2},\ldots ,\psi_{p}$ and $\psi_{1,*},\psi_{2,*},\ldots ,\psi_{p,*}$, the eigenvalues of Ψ and $\Psi_{*}$ respectively since both these matrices are functions of the mixing variable *t*.

### 3.2. Exact Expression for Case 2

An exact expression not consisting of zonal- or Hayakawa polynomials for the capacity (2) for Case 1 is not feasible, due to the intractability of the function ${}_{0}{\tilde{F}}_{1}\left(n;t^{2}\Delta \Sigma^{-1}E\Lambda E^{H}\right)$ in (8). However, an exact expression can be derived for Case 2. For this expression, the computable form for the hypergeometric function of two complex matrix arguments from [21] is exploited. Thus, from (10):

(17) $$\begin{array}{ccc} & & f_{unc}\left(\Lambda \right)\\ & = & \frac{\pi^{p\left(p-1\right)}\det{\left(\Lambda \right)}^{n-p}}{{\tilde{\Gamma }}_{p}\left(p\right){\tilde{\Gamma }}_{p}\left(n\right)}\prod_{k<l}^{p}{\left(\lambda_{k}-\lambda_{l}\right)}^{2}\int_{R^{+}}t^{np}etr\left(-t\left(\Delta +\Lambda \right)\right)\\ & & \times \int_{E\in U\left(p\right)}{\;}_{0}{\tilde{F}}_{1}\left(n;t^{2}\Delta E\Lambda E^{H}\right)dEk\left(t\right)dt\\ & = & \frac{\pi^{p\left(p-1\right)}\det{\left(\Lambda \right)}^{n-p}}{{\tilde{\Gamma }}_{p}\left(p\right){\tilde{\Gamma }}_{p}\left(n\right)}\prod_{k<l}^{p}{\left(\lambda_{k}-\lambda_{l}\right)}^{2}\int_{R^{+}}t^{np}etr\left(-t\left(\Delta +\Lambda \right)\right)\\ & & \times {\;}_{0}{\tilde{F}}_{1}\left(n;t\Omega ,t\Lambda \right)k\left(t\right)dt. \end{array}$$

Ref. [21] enables us to express the function ${}_{0}{\tilde{F}}_{1}\left(n;t\Omega ,t\Lambda \right)$ as follows:

(18) $${}_{0}{\tilde{F}}_{1}\left(n;t\Omega ,t\Lambda \right)=\frac{\det\left({\;}_{0}F_{1}\left(n-p+1,t^{2}\mu_{i}\lambda_{j}\right)\right)}{{\left(\pi t\right)}^{\frac{p\left(p-1\right)}{2}}\prod_{k<l}^{p}\left(\lambda_{k}-\lambda_{l}\right)\prod_{k<l}^{p}\left(\mu_{k}-\mu_{l}\right)}\frac{{\tilde{\Gamma }}_{p}\left(p\right){\tilde{\Gamma }}_{p}\left(n\right)}{{\left(\left(n-p\right)!\right)}^{p}}$$

where ${}_{0}F_{1}\left(\cdot ;\cdot \right)$ denotes the confluent hypergeometric function of scalar argument and $\det\left({}_{0}F_{1}\left(\cdot ;\cdot \right)\right)$ denotes the determinant of a matrix with this confluent hypergeometric function of scalar argument as entries. Please note that ${}_{0}F_{1}\left(s+1;x\right)=s!x^{-\frac{s}{2}}I_{s}\left(2\sqrt{x}\right)$ and that $I_{s}\left(2\sqrt{x}\right)=\sum_{k=0}^{\infty }\frac{1}{k!\Gamma \left(s+k+1\right)}{\left(\frac{2\sqrt{x}}{2}\right)}^{s+2k}$ where $\Gamma \left(\cdot \right)$ denotes the usual gamma function (see [20], p. 919, eq. 8.445). Therefore

(19) $${}_{0}F_{1}\left(n-p+1,t^{2}\mu_{i}\lambda_{j}\right)=\left(n-p\right)!{\left(t^{2}\mu_{i}\lambda_{j}\right)}^{-\frac{n-p}{2}}\sum_{k=0}^{\infty }\frac{1}{k!\Gamma \left(n-p+k+1\right)}{\left(t\sqrt{\mu_{i}\lambda_{j}}\right)}^{n-p+2k}.$$

Using these expressions, it is theoretically possible to numerically solve for the capacity for any *p* by taking the expectation of (2) using the above for the pdf. Of course, this would prove computationally intensive, as the determinant of a (potentially) high dimensional matrix is an expensive operator—in addition to this, the entries of this determinant follow from (19), and would still require the marginal distribution of $\lambda_{1}$ to be numerically determined to evaluate (2). For particular insight into the theoretical framework in this case, assume that p=2:

(20) $$\begin{array}{ccc} & & \det\left({\;}_{0}F_{1}\left(n-1,t^{2}\mu_{i}\lambda_{j}\right)\right)\\ & = & \left|\begin{array}{cc} {\;}_{0}F_{1}\left(n-1,t^{2}\mu_{1}\lambda_{1}\right) & {\;}_{0}F_{1}\left(n-1,t^{2}\mu_{1}\lambda_{2}\right)\\ {\;}_{0}F_{1}\left(n-1,t^{2}\mu_{2}\lambda_{1}\right) & {\;}_{0}F_{1}\left(n-1,t^{2}\mu_{2}\lambda_{2}\right) \end{array}\right|\\ & = & {\;}_{0}F_{1}\left(n-1,t^{2}\mu_{1}\lambda_{1}\right){\;}_{0}F_{1}\left(n-1,t^{2}\mu_{2}\lambda_{2}\right)-{\;}_{0}F_{1}\left(n-1,t^{2}\mu_{1}\lambda_{2}\right){\;}_{0}F_{1}\left(n-1,t^{2}\mu_{2}\lambda_{1}\right)\\ & = & \left(n-2\right)!{\left(t^{2}\mu_{1}\lambda_{1}\right)}^{-\frac{n-2}{2}}\sum_{k=0}^{\infty }\frac{1}{k!\Gamma \left(n-2+k+1\right)}{\left(t\sqrt{\mu_{1}\lambda_{1}}\right)}^{n-2+2k}\left(n-2\right)!{\left(t^{2}\mu_{2}\lambda_{2}\right)}^{-\frac{n-2}{2}}\\ & & \times \sum_{m=0}^{\infty }\frac{1}{m!\Gamma \left(n-2+m+1\right)}{\left(t\sqrt{\mu_{2}\lambda_{2}}\right)}^{n-2+2m}\\ & & -\left(n-2\right)!{\left(t^{2}\mu_{1}\lambda_{2}\right)}^{-\frac{n-2}{2}}\sum_{k=0}^{\infty }\frac{1}{k!\Gamma \left(n-2+k+1\right)}{\left(t\sqrt{\mu_{1}\lambda_{2}}\right)}^{n-2+2k}\\ & & \times \left(n-2\right)!{\left(t^{2}\mu_{2}\lambda_{1}\right)}^{-\frac{n-2}{2}}\sum_{m=0}^{\infty }\frac{1}{m!\Gamma \left(n-2+m+1\right)}{\left(t\sqrt{\mu_{2}\lambda_{1}}\right)}^{n-2+2m}\\ & = & {\left(\left(n-2\right)!\right)}^{2}\sum_{k=0}^{\infty }\sum_{m=0}^{\infty }\frac{t^{2k+2m}}{k!m!\Gamma \left(n+k-1\right)\Gamma \left(n+m-1\right)}\mu_{1}^{k}\mu_{2}^{m}\left(\lambda_{1}^{k}\lambda_{2}^{m}-\lambda_{2}^{k}\lambda_{1}^{m}\right). \end{array}$$

By substituting (21) into (18) leaves:

(21) $$\begin{array}{ccc} {}_{0}{\tilde{F}}_{1}\left(n;t\Omega ,t\Lambda \right) & = & \frac{\det\left({\;}_{0}F_{1}\left(n-p+1,t^{2}\mu_{i}\lambda_{j}\right)\right)}{\pi t\left(\lambda_{1}-\lambda_{2}\right)\left(\mu_{1}-\mu_{2}\right)}\frac{\pi^{2}\pi^{2}\Gamma \left(n\right)\Gamma \left(n-1\right)}{{\left(\left(n-2\right)!\right)}^{2}}\\ & = & \frac{\pi^{2}\pi^{2}\Gamma \left(n\right)\Gamma \left(n-1\right)}{{\left(\left(n-2\right)!\right)}^{2}\pi t\left(\lambda_{1}-\lambda_{2}\right)\left(\mu_{1}-\mu_{2}\right)}{\left(\left(n-2\right)!\right)}^{2}\\ & & \times \sum_{k=0}^{\infty }\sum_{m=0}^{\infty }\frac{t^{2k+2m}\mu_{1}^{k}\mu_{2}^{m}\left(\lambda_{1}^{k}\lambda_{2}^{m}-\lambda_{2}^{k}\lambda_{1}^{m}\right)}{k!m!\Gamma \left(n+k-1\right)\Gamma \left(n+m-1\right)}\\ & = & \frac{\pi^{3}\Gamma \left(n\right)\Gamma \left(n-1\right)}{\left(\lambda_{1}-\lambda_{2}\right)\left(\mu_{1}-\mu_{2}\right)}\sum_{k=0}^{\infty }\sum_{m=0}^{\infty }\frac{t^{2k+2m}\mu_{1}^{k}\mu_{2}^{m}\left(\lambda_{1}^{k}\lambda_{2}^{m}-\lambda_{2}^{k}\lambda_{1}^{m}\right)}{k!m!\Gamma \left(n+k-1\right)\Gamma \left(n+m-1\right)} \end{array}$$

Thus, after considering (22), (18) is shown to be:

(22) $$\begin{array}{ccc} & & f_{unc}\left(\lambda_{1},\lambda_{2}\right)\\ & = & \frac{\pi^{2}{\left(\lambda_{1}\lambda_{2}\right)}^{n-2}}{{\tilde{\Gamma }}_{2}\left(2\right){\tilde{\Gamma }}_{2}\left(n\right)}{\left(\lambda_{1}-\lambda_{2}\right)}^{2}\int_{R^{+}}t^{2n}etr\left(-t\left(\Delta +\Lambda \right)\right)\;\frac{\pi^{3}\Gamma \left(n\right)\Gamma \left(n-1\right)}{\left(\lambda_{1}-\lambda_{2}\right)\left(\mu_{1}-\mu_{2}\right)}\\ & & \times \sum_{k=0}^{\infty }\sum_{m=0}^{\infty }\frac{t^{2k+2m}\mu_{1}^{k}\mu_{2}^{m}\left(\lambda_{1}^{k}\lambda_{2}^{m}-\lambda_{2}^{k}\lambda_{1}^{m}\right)}{k!m!\Gamma \left(n+k-1\right)\Gamma \left(n+m-1\right)}k\left(t\right)dt\\ & = & \frac{1}{\left(\mu_{1}-\mu_{2}\right)}\sum_{k=0}^{\infty }\sum_{m=0}^{\infty }\frac{\mu_{1}^{k}\mu_{2}^{m}\left(\lambda_{1}^{n-1}\lambda_{2}^{n-2}-\lambda_{1}^{n-2}\lambda_{2}^{n-1}\right)\left(\lambda_{1}^{k}\lambda_{2}^{m}-\lambda_{2}^{k}\lambda_{1}^{m}\right)}{k!m!\Gamma \left(n+k-1\right)\Gamma \left(n+m-1\right)}\\ & & \times \int_{R^{+}}t^{2n+2k+2m}etr\left(-t\left(\Delta +\Lambda \right)\right)k\left(t\right)dt. \end{array}$$

This pdf (23) can also be viewed as a weighted infinite sum with Poisson probabilities:

(23) $$\begin{array}{ccc} & & f_{unc}\left(\lambda_{1},\lambda_{2}\right)\\ & = & \frac{\left(\lambda_{1}^{n-1}\lambda_{2}^{n-2}-\lambda_{1}^{n-2}\lambda_{2}^{n-1}\right)}{\left(\mu_{1}-\mu_{2}\right)}\int_{R^{+}}\sum_{k=0}^{\infty }\sum_{m=0}^{\infty }t^{2n+k+m}etr\left(-t\Lambda \right)\frac{\left(\lambda_{1}^{k}\lambda_{2}^{m}-\lambda_{2}^{k}\lambda_{1}^{m}\right)}{\Gamma \left(n+k-1\right)\Gamma \left(n+m-1\right)}\\ & & \times \left(\frac{{\left(t\mu_{1}\right)}^{k}\exp\left(-t\mu_{1}\right)}{k!}\frac{{\left(t\mu_{2}\right)}^{m}\exp\left(-t\mu_{2}\right)}{m!}\right)k\left(t\right)dt \end{array}$$

with Poisson parameters $t\mu_{1}$ and $t\mu_{2}$. The noncentral aspect of this representation thus corresponds with similarly illustrated noncentral representations: in this case by relying on the noncentral matrix eigenvalues $\mu_{1}$ and $\mu_{2}$ as the parameters of Poisson probabilities, which has been observed across the literature: see for example [22,23], and more recently, [24] in the MIMO environment.

For evaluating the capacity (2), see that

$$\begin{array}{ccc} C & = & \int_{R^{+}}2\log\left[1+\frac{\rho }{2}\lambda_{1}\right]\frac{1}{2!\left(\mu_{1}-\mu_{2}\right)}\sum_{k=0}^{\infty }\sum_{m=0}^{\infty }\frac{\mu_{1}^{k}\mu_{2}^{m}}{k!m!\Gamma \left(n+k-1\right)\Gamma \left(n+m-1\right)}\\ & & \times \int_{R^{+}}\left(\lambda_{1}^{n-1}\lambda_{2}^{n-2}-\lambda_{1}^{n-2}\lambda_{2}^{n-1}\right)\left(\lambda_{1}^{k}\lambda_{2}^{m}-\lambda_{2}^{k}\lambda_{1}^{m}\right)\\ & & \times \int_{R^{+}}t^{2n+2k+2m}etr\left(-t\left(\Delta +\Lambda \right)\right)k\left(t\right)dtd\lambda_{2}d\lambda_{1}\\ & = & \frac{1}{\left(\mu_{1}-\mu_{2}\right)}\sum_{k=0}^{\infty }\sum_{m=0}^{\infty }\frac{\mu_{1}^{k}\mu_{2}^{m}}{k!m!\Gamma \left(n+k-1\right)\Gamma \left(n+m-1\right)}\int_{R^{+}}t^{2n+2k+2m}etr\left(-t\Delta \right)\\ & & \times \int_{R^{+}}\log\left[1+\frac{\rho }{2}\lambda_{1}\right]\exp\left(-t\lambda_{1}\right)\\ & & \times \int_{R^{+}}\left(\lambda_{1}^{n-1}\lambda_{2}^{n-2}-\lambda_{1}^{n-2}\lambda_{2}^{n-1}\right)\left(\lambda_{1}^{k}\lambda_{2}^{m}-\lambda_{2}^{k}\lambda_{1}^{m}\right)\exp\left(-t\lambda_{2}\right)d\lambda_{2}d\lambda_{1}k\left(t\right)dt \end{array}$$

where using the gamma integral relation again leaves

$$\begin{array}{ccc} C & = & \frac{1}{\left(\mu_{1}-\mu_{2}\right)}\sum_{k=0}^{\infty }\sum_{m=0}^{\infty }\frac{\mu_{1}^{k}\mu_{2}^{m}}{k!m!\Gamma \left(n+k-1\right)\Gamma \left(n+m-1\right)}\int_{R^{+}}t^{n+2k+2m}etr\left(-t\Delta \right)\\ & & \times [t^{-\left(m-1\right)}\Gamma \left(n+m-1\right)\int_{R^{+}}\log\left[1+\frac{\rho }{2}\lambda_{1}\right]\exp\left(-t\lambda_{1}\right)\lambda_{1}^{n+k-1}d\lambda_{1}\\ & & -t^{-\left(k-1\right)}\Gamma \left(n+k-1\right)\int_{R^{+}}\log\left[1+\frac{\rho }{2}\lambda_{1}\right]\exp\left(-t\lambda_{1}\right)\lambda_{1}^{n+m-1}d\lambda_{1}\\ & & -t^{-m}\Gamma \left(n+m\right)\int_{R^{+}}\log\left[1+\frac{\rho }{2}\lambda_{1}\right]\exp\left(-t\lambda_{1}\right)\lambda_{1}^{n+k-2}d\lambda_{1}\\ & & +t^{-k}\Gamma \left(n+k\right)\int_{R^{+}}\log\left[1+\frac{\rho }{2}\lambda_{1}\right]\exp\left(-t\lambda_{1}\right)\lambda_{1}^{n+m-2}d\lambda_{1}]k\left(t\right)dt. \end{array}$$

This exact expression for the capacity provides meaningful theoretical insight to the form of the capacity when considering uncorrelated MIMO channels subject to an SMCN propagation channel H, and has the results of [2,12] as a special case for corresponding choices of k(t). The view of (24) solidifies the noncentral consideration for this bivariate case and acts as a "benchmark" of the noncentral representation which is often encountered in the literature.

## 4. Conclusions

In this paper, a motivated argument was presented for the introduction of an SMCN distribution for the underlying model of the propagation matrix H in a MIMO system environment. Not only does this assumption relieve the restriction of normality for the practitioner, but in this case a nonzero mean is also assumed as part of this model. Capacity, an essential measure of information within the MIMO context, relies on the distribution of $H^{H}H$, and this paper investigates the eigenvalue relations of this SMCW distribution, as well as its uncorrelated counterpart. An upper bound for the capacity for both these cases are derived for the case when p=2, which in a MIMO context implies a two transmitter environment and is often employed for dual branch MIMO systems in practice, while considering arbitrary number of receivers *n*. This theoretical framework of the scale mixture assumption for the propagation matrix and the mathematical effect of this assumption in theoretically understanding and quantifying the capacity of such a system enjoys specific conceptual insight.

For higher orders of *p* the complexity of the calculation may be well reached in computational terms, but in a theoretical sense the representations become challenging—even more so because of the nonzero mean assumption: it is essential to note that $\psi_{1},\psi_{2},\ldots ,\psi_{p}$ and $\psi_{1,*},\psi_{2,*},\ldots ,\psi_{p,*}$ are eigenvalues of complex matrices inherently dependent on the scale mixture variable *t*. These challenges are mostly due to the potential high dimensionality of an arbitrary *p*, and the provision of Δ via Ψ or $\Psi_{*}$ in accounting for LOS in a practical consideration. Perhaps this does not facilitate convenient immediate computation for the capacity, but it does give us insight into the theoretical framework of the capacity when assuming $H∼SMCN_{n\times p}\left(M,\Phi ⊗\Sigma ,h\right)$ which remains a meaningful and popular choice for the departure from normality within the statistical arena. In future, one might consider the further investigation of higher orders of *p*, the computational complexity involved with it, and the derivations and interpretations of other measures of information such as types of condition numbers and outage probabilities for models under the key assumptions made in this paper. A refreshing contribution to the literature could also be made by considering complex matrix variate Gaussian mixtures for the underlying distribution, as described in Section 1.

## Data Availability

Not applicable.

## Abbreviations

MIMO	multiple-input-multiple-output
LOS	line-of-sight
CN	complex matrix variate normal
SMCN	scale mixture of complex matrix variate normal
SMCW	scale mixture of complex Wishart
pdf	probability density function

## Appendix A

The statistical characterization of the fading (or, degradation) of the signal between transmitters and receivers in the case of a nonzero M case is described by the Rician distribution. This distribution has its direct roots in the usual CN assumption for H; thus it is necessary to report the equivalent fading distribution in the case where H is assumed to be an SMCN distribution. Suppose *R* is the fading variable; then, under the SMCN assumption, the pdf is given by

(A1) $$f\left(r\right)=\int_{R^{+}}\frac{r}{\sigma^{2}t^{-1}}\exp\left(-\frac{r^{2}+s^{2}}{2\sigma^{2}t^{-1}}\right)I_{0}\left(\frac{rs}{\sigma^{2}t^{-1}}\right)k\left(t\right)dt,r>0$$

where $s^{2},\sigma^{2}>0$ and $I_{v}\left(.\right)$ denotes the modified Bessel function of the first kind [20]. Note in particular that when $s^{2}=0$, then (A1) reduces to the Rayleigh type distribution (or in other words, a Rayleigh type fading scenario) when the assumption of H is that of a zero mean SMCN distribution (see [11]). It is essential to observe that for the specific case when H is a CN candidate (for the specific choice of $k\left(t\right)$) then (A1) reflects the usual Rician fading scenario–see also [10,12]. In addition to this, it is valuable to note the recent consideration of the Rayleigh type model emanating from a scale mixture of normal approach in the work of [25].

## References

[1] Choi S.H. (2007). Severly Fading MIMO Channels. Unpublished. Master’s Thesis.

[2] Ratnarajah T., Vaillancourt R., Alvo M. (2005). Complex random matrices and Rician channel capacity. Probl. Inf. Transm..

[3] Selim B., Alhussein O., Muhaidat S., Karagiannidis G.K., Liang J. (2015). Modeling and analysis of wireless channels via the mixture of Gaussian distribution. IEEE Trans. Veh. Technol..

[4] Alhussein O., Selim B., Assaf T., Muhaidat S., Liang J., Karagiannidis G.K. A generalized mixture of Gaussians for fading channels. Proceedings of the 2015 IEEE 81st Vehicular Technology Conference (VTC Spring).

[5] Chu K.C. (1973). Estimation and decision for linear systems with elliptical random processes. IEEE Trans. Autom. Control..

[6] Andrews D.F., Mallows C.L. (1974). Scale mixtures of normal distributions. J. R. Stat. Soc. Ser. (Methodol.).

[7] West M. (1987). On scale mixtures of normal distributions. Biometrika.

[8] Provost S.B., Cheong Y.H. (2002). The distribution of Hermitian quadratic forms in elliptically contoured random vectors. J. Stat. Plan. Inference.

[9] Choi S.H., Smith P., Allen B., Malik W.Q., Shafi M. Severely fading MIMO channels: Models and mutual information. Proceedings of the 2007 IEEE International Conference on Communications.

[10] Ferreira J.T., Bekker A. (2019). A unified complex noncentral Wishart type distribution inspired by massive MIMO systems. J. Stat. Distrib. Appl..

[11] Ferreira J.T., Bekker A., Arashi M. (2020). Advances in Wishart-type modelling of channel capacity. Revstat-Stat. J..

[12] Ferreira J.T., Bekker A. (2020). Weighted condition number distributions emanating from complex noncentral Wishart type matrices. Computational and Methodological Statistics and Biostatistics.

[13] Ollila E., Eriksson J., Koivunen V. (2010). Complex elliptically symmetric random variables—generation, characterization, and circularity tests. IEEE Trans. Signal Process..

[14] Kang M., Alouini M.S. (2006). Capacity of MIMO Rician channels. IEEE Trans. Wirel. Commun..

[15] McKay M.R., Collings I.B. (2005). General capacity bounds for spatially correlated Rician MIMO channels. IEEE Trans. Inf. Theory.

[16] Khatri C. (1969). Non-central distributions of *i*^th^ largest characteristic roots of three matrices concerning complex multivariate normal populations. Ann. Inst. Stat. Math..

[17] Matthaiou M., Laurenson D.I., Wang C.X. (2009). On analytical derivations of the condition number distributions of dual non-central Wishart matrices. IEEE Trans. Wirel. Commun..

[18] James A.T. (1964). Distributions of matrix variates and latent roots derived from normal samples. Ann. Math. Stat..

[19] Bain L.J., Engelhardt M. (1987). Introduction to Probability and Mathematical Statistics.

[20] Gradshteyn I.S., Ryzhik I.M. (2014). Table of Integrals, Series, and Products.

[21] Gross K.I., Richards D.S.P. (1989). Total positivity, spherical series, and hypergeometric functions of matrix argument. J. Approx. Theory.

[22] Patnaik P. (1949). The non-central *χ*^2^-and F-distribution and their applications. Biometrika.

[23] Ferreira J.T., Bekker A., Arashi M. (2016). Bivariate noncentral distributions: An approach via the compounding method. S. Afr. Stat. J..

[24] Bekker A., Ferreira J.T. (2018). Bivariate gamma type distributions for modeling wireless performance metrics. Stat. Optim. Inf. Comput..

[25] Saeedzarandi M., Nezamabadi-pour H., Jamalizadeh A. (2020). Dual-Tree complex wavelet coefficient magnitude modeling using scale mixtures of Rayleigh distribution for image denoising. Circuits Syst. Signal Process..

